# The efficacy of cervical spine phantoms for improving resident proficiency in performing ultrasound-guided cervical medial branch block: A prospective, randomized, comparative study

**DOI:** 10.1097/MD.0000000000013765

**Published:** 2018-12-21

**Authors:** So Young Kwon, Jong-Woan Kim, Min Ji Cho, Abdullah Hussain Al-Sinan, Yun-Joung Han, Young Hoon Kim

**Affiliations:** a Department of Anesthesiology and Pain Medicine, St. Vincent's Hospital, College of Medicine, The Catholic University of Korea, Suwon; b Department of Anesthesiology and Pain Medicine, Seoul St. Mary's Hospital, College of Medicine, The Catholic University of Korea, Seoul, Republic of Korea; c Department of Anesthesiology and Pain Medicine, Ministry of Health, Qatif Central Hospital, Alawjam, Saudi Arabia.

**Keywords:** cervical medial branch block, improved proficiency, phantom, resident training, simulation, ultrasound-guided procedure

## Abstract

**Background:**

Few studies have been conducted on the utility of cervical spine phantoms for practicing cervical procedures. Here, we describe a simple method for creating a cervical spine phantom and investigate whether the use of a gelatin-based phantom is associated with improved proficiency in performing ultrasound-guided cervical medial branch block.

**Methods:**

A cervical spine phantom was prepared using a cervical spine model immersed in a mixture of gelatin and psyllium husk. In total, 27 participants, inexperienced in spinal ultrasonography, were enrolled and allocated to 1 of 2 groups (training group, n = 18; control group, n = 9). All participants were tested (test-1) following an introductory course of basic ultrasonography. Participants in the control group were tested again after 1 week (test-2). Those in the training group received a further individual 3-hour training session, and were tested again after 1 week (test-2).

**Results:**

The mean performance score in test-1 was 62.5 ± 10.1 points in the training group and 62.3 ± 4.1 points in the control group [95% confidence interval (95% CI) −5.5 to 5.8; *P* = .954]. In test-2, the mean score was 86.8 ± 6.5 points and 59.9 ± 4.4 points in the training and control groups, respectively (95% CI 21.9–31.8; *P* < .001). The mean time required to complete test-1 was 84.6 ± 26.6 seconds in training group and 90.7 ± 43.9 seconds in the control group (95% CI −34.0 to 21.7; *P* = .653); in test-2, the time required was 56.6 ± 27.9 and 91.2 ± 43.8 seconds (95% CI −63.0 to −6.2; *P* = .019), respectively. Interobserver reliability showed excellent agreement based on the intraclass correlation coefficient, and moderate to almost perfect agreement by kappa statistics.

**Conclusion:**

Training using a gelatin-based cervical spine phantom helps novices acquire the skills necessary to perform ultrasound-guided cervical medial branch blocks.

## 1 Introduction

Ultrasound is increasingly used for diagnosis and treatment in the field of pain management. Ultrasound avoids exposure to radiation hazards, allows the target and needle to be visualized in real time, and permits the spread of injectate to be identified. Spinal interventions, such as cervical medial branch blocks, are commonly performed under ultrasound or fluoroscopic guidance.^[1,2]^ Beginners can acquire ultrasound scanning skills through lectures or workshops. To learn how to execute ultrasound-guided procedures, novices can participate in cadaver workshops or obtain real-time experience by carefully conducting the procedures on patients. However, the enormous cost of cadavers, the need for cooperation with the anatomy department, and time constraints create practical difficulties when training beginners. In particular, it is not ethically desirable for inexperienced individuals to practice needle placement on patients. Even if novices successfully acquire knowledge on sonoanatomy and ultrasonography through lectures and workshops, it is common for them to fail to visualize the needle during insertion while performing ultrasound-guided regional anesthesia.^[3]^ An expert committee determined that the level of difficulty of cervical medial branch block procedures was “level III (advanced).”^[4]^ The spinal cord injury has been reported as a serious complication.^[5]^ Therefore, a great deal of practice is required to master ultrasound-guided cervical medial branch block. Despite the rapid growth in ultrasound usage, few curricula or programs for ultrasound-guided needle-base procedures have been reported.^[6–9]^

Commercially manufactured phantoms can be used to practice needle placement easily and safely.^[10,11]^ Patient safety is assured, multiple uses are possible, and models with a variety of specific anatomical features can be selected. This leads to improved trainee confidence and performance. Nevertheless, such models are costly. Phantoms manufactured with specific anatomical features can also be more expensive than other models. Gelatin-based lumbar spine phantoms have recently been developed.^[12–14]^ A recent report discussed the efficacy of lumbosacral spine phantoms in improving resident proficiency in ultrasound-guided lumbar facet joint injections and medial branch blocks.^[13]^ However, few studies have discussed how to create a gelatin-based cervical spine phantom, or how to improve novices’ skills in cervical interventions.^[15,16]^

The present study aimed to develop an easily fabricated gelatin-based cervical spine phantom, and to evaluate the utility of this phantom for improving beginners’ proficiency in performing ultrasound-guided cervical medial branch blocks.

## 2 Methods

### 2.1 Participants

**Figure 1:**
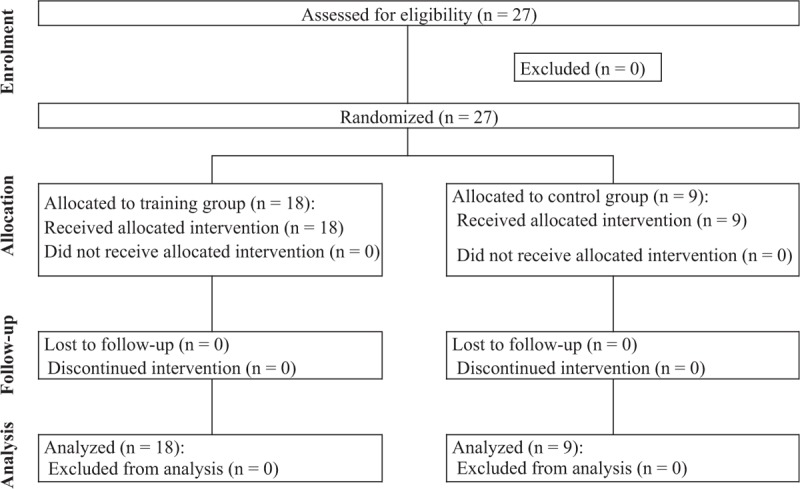
Flow chart according to Consolidated Standards of Reporting Trials statement (CONSORT).

The study protocol was reviewed and approved by the Institutional Review Board of Seoul St. Mary's Hospital, Catholic University (IRB No. KC16OISI0927). Twenty-seven participants with no experience in spinal ultrasonography were enrolled in this study. Participants provided informed consent for review of their test scores and were allocated to 1 of 2 groups using a computer-generated random sequence. Before group allocation, envelopes containing the programs were numbered sequentially and sealed. The sealed envelopes were opened by an investigator blinded to the trainees’ assessment. One group was composed of residents who were not to be trained using a cervical spine phantom (control group, n = 9), while the other group comprised residents who were to be trained using a phantom (training group, n = 18) (Fig. 1).

### 2.2 The phantom

**Figure 2:**
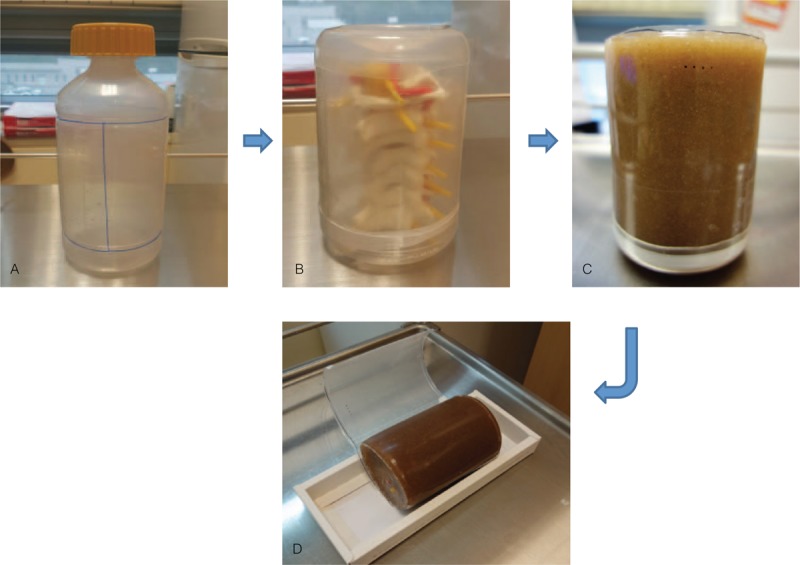
The process of fabricating the cervical spine phantom. (A) Blue lines indicate segments to be cut.

A cervical spine phantom was made with a cervical spine model of C1 to C7 (Cervical Spinal Column A72; 3B Scientific, Inc., Hamburg, Germany) embedded in a mixture of gelatin (bovine collagen hydrolysate; Geltech, Busan, Korea) and psyllium husk (Whole Psyllium Husks; NOW Foods, Inc., Bloomingdale, IL). The middle section of an empty 1 L plastic bottle for sterile normal saline (diameter ∼9 cm and height ∼13 cm) was cut to simulate a human neck (Fig. 2A). The cut bottom of the plastic bottle was used to cover the end of this cylindrical plastic container. Teflon tape made from tetrafluoroethylene monomers was wrapped around the lower part of the cylindrical plastic structure to prevent leakage of the melted mixture of gelatin and psyllium. The adult-sized cervical spine model, which was articulated and had artificial neuraxial structures (structures such as the dura and ligaments were not included), was encased in the cylindrical plastic container. The metal part of a cervical spine model was removed in advance (Fig. 2B). The gelatin mixture was produced by dissolving 120 g of gelatin and 36 g of psyllium husk into about 1 L of hot tap water (≥170°F; Fig. 2C).^[12–14]^ The psyllium husk was added to simulate the appearance of soft tissue and the mixture was poured into the container. The cervical spine phantom was kept under refrigeration overnight to harden the mixture (Fig. 2D). The total cost, including the gelatin, psyllium, empty plastic bottle, and cervical spine model, was about $130. We made 54 phantoms. Each participant used 2 phantoms for the tests and training. Scanning and injection practice leaves needle-track marks in the phantom, which could affect subsequent ultrasound scans. To overcome this issue, the gelatin and psyllium mixture was completely redissolved in the microwave. After keeping the phantom refrigerated overnight, the phantom could be reused for scanning and injection practice.

### 2.3 Ultrasound equipment

The ultrasound-guided procedures were performed with an ultrasound device (Edge; SonoSite, Bothell, WA) with a linear transducer at 5 to 13 MHz. We used a disposable 24-gauge, 60 mm needle.

### 2.4 Curriculum and tests

**Figure 3:**
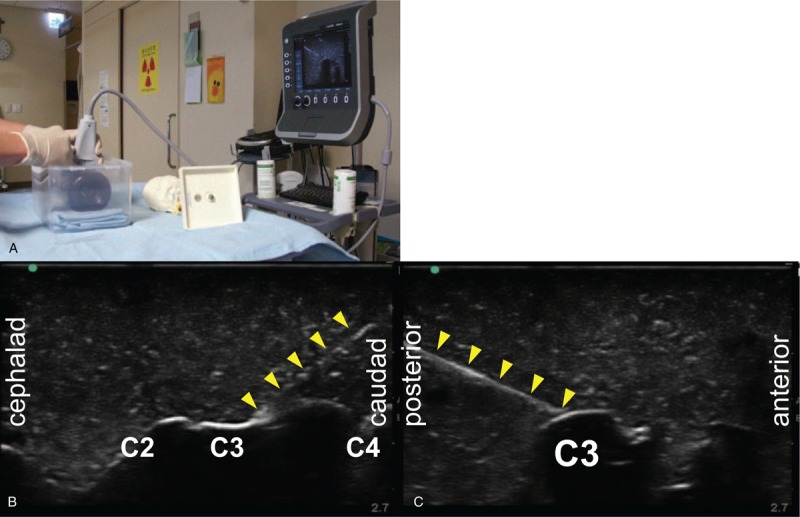
Ultrasound-guided cervical medial branch block using a cervical spine phantom. (A) A specialist demonstrates how to perform the procedure using the phantom. The phantom is placed in front of the specialist with the ultrasound transducer firmly grabbed using the first three fingers while resting the 4th and 5th fingers on the surface of the phantom to give more stability. The transducer is then rotated to obtain an axial view of the lower cervical spine. (B) Coronal scan. The needle tip is aimed approximately at the mid-point of the interarticular pillar of C3. (C) Axial scan. Arrowheads indicate needles.

All participants watched a 15-minute video lecture providing a theoretical introduction to the basics of ultrasound, ultrasound transducers, in-plane and out-of-plane approaches, the anatomical structures of the cervical spine, and the cervical medial branch block procedure. Next, an ultrasound specialist (one of the authors) described how to handle the transducer and perform ultrasound-guided cervical medial branch blocks using the phantom (Fig. 3).

All participants were tested after this basic introduction (test-1). Participants in the control retook the test after 1 week (test-2). Those in the training group were individually trained for more than 3 hours and tested again after 1 week (test-2). The tests were scored by 2 independent ultrasound specialists with more than 9 years of experience in spinal ultrasonography.

As there is no specific scoring system for evaluating ultrasound-guided block, we used that described in the study of Kwon et al.^[13]^ Their scoring system encompasses the following 6 domains: ergonomics, proper handling of the transducer, sonographic localization of the lesion, insertion of the needle at an appropriate distance from the transducer, visualization of the needle throughout the procedure, and proper placement of the needle in the target lesion. All participants were asked to locate the right articular processes from C2 to C7 sequentially on a coronal scan obtained under ultrasonography. If the process took less than 60 seconds, the test score was 2 points, and if it took more than 60 seconds, the score was 0 points (“score A”). Then, participants conducted right C3 and C4 medial branch blocks under the coronal view and the axial view with in-plane approaches, and checked the location of the needle using fluoroscopy. Two evaluators rated the 6 items of the scoring system on a scale ranging from 1 to 4 for each of the 4 blocks, and the sum of the scores and mean values were calculated (“score B”). Each participant was scored on a 100-point scale according to the following formula: score = (score A + score B)/26 × 100.

### 2.5 Evaluation of primary outcome

Following the procedures, we scaled and compared the average performance scores of the 2 assessors, as discussed above.

### 2.6 Evaluation of secondary outcomes

The time from when the transducer contacted the skin to when the position of the needle was fluoroscopically checked was defined as the procedure time. We compared the mean scores of each item of the scoring system between test-1 and test-2 to ascertain which scores significantly improved after using the cervical spine phantom. The participants in the training group were asked to rate their own proficiency before and after the training, and to rate the extent to which the training program improved their proficiency on scale ranging from 0 (unsatisfactory) to 10 (perfect).

### 2.7 Statistical analysis

Before the full study, we performed a pilot study with residents who participated in the introductory program. The mean performance score ± standard deviation (SD) was estimated to be 58.9 ± 12.0 points. Eight participants were required in the control group and 16 in the training group to detect a 15-point increase in the performance score, assuming α = 0.05 (2-tailed) and β = 0.2 (80% power) with a 1:2 allocation ratio. Assuming account a dropout rate of 10%, 9 participants were allocated to the control group and 18 to the training group. Continuous data were tested for normality using the Shapiro–Wilk test. Normally distributed data and non-normally distributed data are presented as mean ± SD and median (range), respectively. To compare performance scores, self-rating scores for proficiency and procedure time between the 2 tests, the normally distributed data were analyzed using paired *t* tests; non-normally distributed data were compared using the Wilcoxon signed-rank test. For comparisons between 2 groups, the normally distributed data were analyzed using Student *t* test and the non-normally distributed data were compared using the Mann–Whitney *U* test. The self-rating scores associated with the extent to which the training course improved the trainees’ proficiency were expressed as the medians, 25th and 75th percentiles, and maximum and minimum values using a boxplot. The intraclass correlation coefficient (ICC) and kappa statistics were used to assess the interobserver reliability of the scoring system. We adapted a scale reported previously for the interpretation of ICC: ICC values of less than 0.40 represent poor reproducibility, values in the range of 0.40 to 0.75 represent fair-to-good reproducibility, and values greater than 0.75 represent excellent reproducibility.^[17]^ In accordance with the suggestion by Landis and Koch,^[18]^ the extent of agreement was described as follows: kappa values of 0 to 0.2 indicate slight agreement, 0.21 to 0.4 fair agreement, 0.41 to 0.60 moderate agreement, 0.61 to 0.80 substantial agreement, and 0.81 or greater almost perfect agreement. *P* values < .05 were considered statistically significant. Data were analyzed using SPSS software (ver. 18.0; SPSS Inc., Chicago, IL).

## 3 Results

### 3.1 Performance scores and times

**Figure 4:**
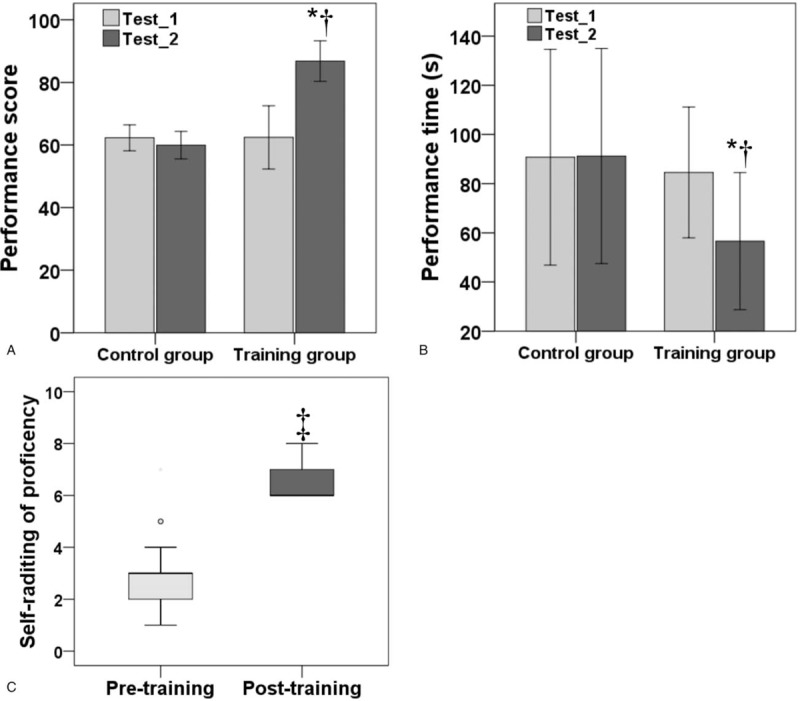
Comparison of outcomes. (A) Performance scores. (B) Performance times. (C) Self-rating scores for proficiency. ^∗^*P* < .001 compared with test 1; ^†^*P* < .001 compared with control group; ^‡^*P* < .001 compared with pre-training scores.

The mean performance score in test-1 was 62.5 ± 10.1 points in the training group and 62.3 ± 4.1 points in the control group [95% confidence interval (95% CI) −5.5 to 5.8; *P* = .954]; in test-2, it was 86.8 ± 6.5 points and 59.9 ± 4.4 points, respectively (95% CI 21.9 to 31.8; *P* < .001) (Fig. 4A). The mean performance time for test-1 was 84.6 ± 26.6 seconds in the training group and 90.7 ± 43.9 seconds in the control group (95% CI −34.0 to 21.7; *P* = .653). In test-2, the mean performance time was 56.6 ± 27.9 and 91.2 ± 43.8 seconds for the control and training group, respectively (95% CI −63.0 to −6.2; *P* = .019) (Fig. 4B). In the control group, no item on the scoring system showed significant improvement between the 2 tests. In the training group, all items except “ergonomics” (*P* = .453) improved significantly (*P* < .001).

### 3.2 Self-rating of proficiency

In the training group, the median self-rating score for proficiency was 3.0 (1.0–7.0) points before training and 6.0 (6.0–8.0) after training (*P* < .001; Fig. 4C). The mean self-rating score associated with the extent to which the training course improved the trainees’ proficiency was 7.0 ± 1.3 points.

### 3.3 Inter-observer reliability

The interobserver agreement regarding the assessment of the scoring system is shown in terms of ICC and kappa statistics in Table 1. The ICC values in the range 0.822 to 0.993 indicated excellent agreement. The kappa values ranged from 0.557 to 0.973, indicating moderate to almost perfect agreement.

**Table 1:**
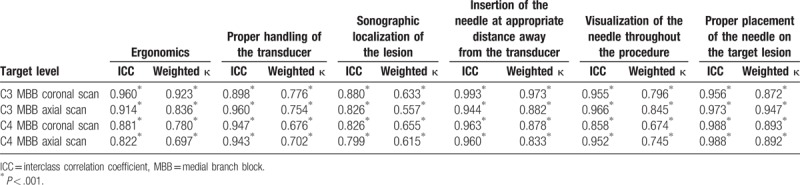
Interobserver reliability.

## 4 Discussion

To the best of our knowledge, there is no study to assess the efficacy of cervical spine phantoms for improving novices’ proficiency in performing cervical interventions. Our findings show that a training curriculum incorporating a gelatin-based cervical spine phantom significantly improved novices’ procedure proficiency and time. On the basis of participants’ subjective evaluations, self-rated proficiency scores increased significantly after training. Consequently, this study showed that the trainees who practiced ultrasound-guided cervical medial branch blocks using a gelatin-based cervical spine phantom outperformed those who did not.

Experts recommend several measures to achieve proficiency during ultrasonography: practicing ultrasound scanning techniques and learning sonoanatomy by imaging oneself and colleagues; practicing needle insertion technique using simulators, phantoms, and cadavers; and conducting needle placement on patients under the supervision of experienced individuals.^[4]^ The most common error of beginners performing ultrasound-guided regional anesthesia is failure to visualize the needle during insertion.^[3]^ As it is not ethically desirable for novices to practice this procedure on patients, it is recommended that they practice ultrasound-guided injection and ultrasound scanning using phantoms.^[4,13,19]^ Furthermore, ultrasound-guided cervical medial branch block should also be practiced using phantoms, as this procedure has a difficulty of level III (advanced) owing to the presence of critical structures in neck.^[4]^

Appropriate phantoms should be selected based on cost, availability, degree of tactile feedback, and the specific skill to be practiced. A water phantom is inexpensive, easy to use, and allows the needle and target to be clearly visualized. However, it is not suitable for practicing needle injection, as it provides no tactile feedback. Commercially available phantoms are widely used in training courses because they provide better tactile feedback. However, such phantoms are expensive, and different phantoms need to be purchased depending on the type of procedure to be performed. A meat phantom provides some tactile feedback and anatomic structure, and enables trainees to simulate local anesthetic injection and dissection. Cadavers are not readily available without cooperation from an anatomy department, and are costly to use. However, if cadavers are available, they provide favorable imaging characteristics, tactile feedback similar to living human tissue, and opportunities to inject local anesthetic and dissect the target tissue. Gelatin-based phantoms are inexpensive, simple to produce, and provide satisfactory tactile feedback. Phantoms simulating specific body parts can be simulated by immersing structures in a gelatin solution.^[11,13,16,19]^ To simulate human soft tissue, materials such as flour, mutacil, graphite powder, and psyllium husk can be added to the solution. In the present study, the outer container for our cervical spine phantom was removed before use; we therefore used a gelatin to psyllium husk ratio of 3.3:1 to increase durability and prevent damage from ultrasound transducers.^[13,20]^

Recent studies have evaluated the reliability and validity of a task-specific checklist and global rating scale used for assessment of ultrasound-guided regional anesthesia competency.^[21–23]^ On the basis of a simulation model, the global rating scale was able to differentiate novices from experienced physicians, while the checklist was not.^[21]^ These tools were developed to assess procedure skills, but may be too complex for practical use. The checklist may also require modification to evaluate specific procedures.

There are few data on global rating scales that optimally evaluate ultrasound-guided spine procedure using inanimate phantoms. Ball et al^[24]^ measured the procedure time, and the number of needle sticks and needle redirections, to evaluate the effectiveness of the simulation technique. Michalek et al^[25]^ used success rate and time required to perform a procedure to assess imaging quality and the block technique. However, the factors above are not sufficient to fully assess the quality of a procedure. In a recent study by Brascher et al,^[26]^ participants were asked to provide responses on a scale ranging from 1 (excellent) to 6 (unsatisfactory) to evaluate the curriculum, which had the drawback of being highly subjective. Therefore, we used a scoring system modeled after the assessment tool used in Kwon et al,^[13]^ which consists of several items based on the checklist and the global rating scale.^[22]^ The scoring system showed strong interobserver reliability based on ICC and kappa statistics. In the training group, all items on the checklist (except “Ergonomics”) improved significantly between test-1 and test-2, whereas no such improvement occurred in the control group. It is possible that ergonomic skills were already at a high level because of the introductory teaching video provided before training. In general, these results showed that the training regime was effective in improving procedural skill.

Only a few studies have fabricated cervical spine phantoms.^[15,16,26]^ In a study by Lerman et al,^[15]^ a phantom for cervical transforaminal injection was developed. Although they simulated vertebral arteries in the spine phantom, the use of polyvinyl-chloride plastic material necessitated a well-ventilated area, as well as the use of protective eyewear and gloves for safety. In a report by van Eerd et al,^[16]^ a plastic cervical spine was placed in a pre-made polycarbonate cylinder that was relatively difficult to fabricate. Although it was similar to the phantom used in the present study, it did not simulate soft tissue with materials such as psyllium husk; furthermore, the authors did not evaluate its utility for education and training. Brascher et al^[26]^ also created a cervical phantom to simulate stellate ganglion block. They used plaster to express the transverse process of cervical vertebrae seen in the anterior neck. However, their phantom is not appropriate for cervical medial branch blocks. In the present study, we used an empty 1-L plastic bottle as the outer container for the phantom. Such bottles are cheap, and easy to obtain and handle. Furthermore, the cervical spine model can be heated and reset, permitting it to be reused as many times as needed.

The present study had several limitations. First, there was the potential for imparting false confidence to novices during training, as the gelatin-based phantom was simpler and easier to visualize than human ultrasound images. Second, we evaluated only in-plane needle injections, to allow scanning of both the needle shaft and the tip. As out-of-plane approaches may be required during cervical medial branch block,^[27]^ the suitability of this spine phantom for training on out-of-plane approaches should be assessed. Third, although the spine model is adult-sized, the plastic saline bottle is smaller than typical human necks, making the procedure easier than in actual clinical practice. Fourth, it was not easy to simulate the difficulty of the procedure with respect to the structure of the shoulder at the C7 region, a region in which accuracy is lower than in other areas.^[27]^ Practitioners should also take care during cervical spine interventions, as no phantom developed to date accurately simulates periforaminal vessels, including ascending cervical or radicular arteries.^[15,28,29]^ Finally, further trials are required to demonstrate that the training curriculum described herein improves beginners’ proficiency in clinical settings.

In conclusion, this study shows that training using a gelatin-based cervical spine phantom helps beginners improve the skills needed to perform ultrasound-guided cervical medial branch blocks.

## Acknowledgment

The authors wish to acknowledge the financial support of the Catholic Medical Center Research Foundation made in the program year of 2016.

## Author contributions

**Conceptualization:** So Young Kwon, Young Hoon Kim.

**Data curation:** Yun-Joung Han, Young Hoon Kim.

**Formal analysis:** So Young Kwon.

**Investigation:** Jong-Woan Kim, Min Ji Cho, Abdullah Hussain Al-Sinan.

**Methodology:** Jong-Woan Kim, Min Ji Cho, Abdullah Hussain Al-Sinan.

**Supervision:** Yun-Joung Han.

**Writing – original draft:** So Young Kwon, Young Hoon Kim.

**Writing – review & editing:** So Young Kwon, Young Hoon Kim.

Young Hoon Kim orcid: 0000-0001-6685-1244.
